# Complete reversal of epithelial to mesenchymal transition requires inhibition of both ZEB expression and the Rho pathway

**DOI:** 10.1186/1471-2121-10-94

**Published:** 2009-12-21

**Authors:** Shreyas Das, Bryan N Becker, F Michael Hoffmann, Janet E Mertz

**Affiliations:** 1 McArdle Laboratory for Cancer Research, University of Wisconsin School of Medicine and Public Health, 1400 University Ave, Madison, Wisconsin 53706, USA; 2 Laboratory of Genetics, University of Wisconsin School of Medicine and Public Health, 425-G Henry Mall, Madison, Wisconsin 53706, USA; 3 Department of Medicine, University of Wisconsin School of Medicine and Public Health, 600 Highland Avenue, Madison, Wisconsin 53792, USA

## Abstract

**Background:**

Epithelial to Mesenchymal Transition (EMT) induced by Transforming Growth Factor-β (TGF-β) is an important cellular event in organogenesis, cancer, and organ fibrosis. The process to reverse EMT is not well established. Our purpose is to define signaling pathways and transcription factors that maintain the TGF-β-induced mesenchymal state.

**Results:**

Inhibitors of five kinases implicated in EMT, TGF-β Type I receptor kinase (TβRI), p38 mitogen-activated protein kinase (p38 MAPK), MAP kinase kinase/extracellular signal-regulated kinase activator kinase (MEK1), c-Jun NH-terminal kinase (JNK), and Rho kinase (ROCK), were evaluated for reversal of the mesenchymal state induced in renal tubular epithelial cells. Single agents did not fully reverse EMT as determined by cellular morphology and gene expression. However, exposure to the TβRI inhibitor SB431542, combined with the ROCK inhibitor Y27632, eliminated detectable actin stress fibers and mesenchymal gene expression while restoring epithelial E-cadherin and Kidney-specific cadherin (Ksp-cadherin) expression. A second combination, the TβRI inhibitor SB431542 together with the p38 MAPK inhibitor SB203580, was partially effective in reversing EMT. Furthermore, JNK inhibitor SP600125 inhibits the effectiveness of the TβRI inhibitor SB431542 to reverse EMT. To explore the molecular basis underlying EMT reversal, we also targeted the transcriptional repressors ZEB1 and ZEB2/SIP1. Decreasing ZEB1 and ZEB2 expression in mouse mammary gland cells with shRNAs was sufficient to up-regulate expression of epithelial proteins such as E-cadherin and to re-establish epithelial features. However, complete restoration of cortical F-actin required incubation with the ROCK inhibitor Y27632 in combination with ZEB1/2 knockdown.

**Conclusions:**

We demonstrate that reversal of EMT requires re-establishing both epithelial transcription and structural components by sustained and independent signaling through TβRI and ROCK. These findings indicate that combination small molecule therapy targeting multiple kinases may be necessary to reverse disease conditions.

## Background

Epithelial to Mesenchymal Transition (EMT) is an extreme form of cellular plasticity defined by loss of epithelial cell morphology, dissociation of cell-cell contacts, reduction in proteins mediating cell-cell contacts, remodeling of the actin cytoskeleton, *de novo *expression of α-smooth muscle actin (α-SMA), and acquisition of mesenchymal cell shape [1-4]. During EMT, cells diminish epithelial gene expression and acquire mesenchymal gene expression [5]. Cortical actins, the actin filament bundles below the plasma membrane, reorganize or are lost, while stress fibers comprising F-actin are gained. In normal development, EMT has been associated with processes in gastrulation, heart formation, palate formation, and Mullerian tract regression [4]. In disease states, EMT has been exploited in both cancer and organ fibrosis. The mortality in human cancers is caused by primary tumor cells that have undergone oncogenic EMT and metastasized to other organs. Other diseases, such as end-state organ failure by fibrosis, are caused by repeated and sustained infliction of EMT. Thus, understanding the cellular mechanisms to reverse EMT is of great importance.

The TGF-β signaling pathway is considered a good target for EMT reversal because it is a key mediator of fibrosis and facilitator of metastasis [3,6]. TGF-β induces EMT by both Smad-dependent and -independent signaling events [4,7,8]. TGF-β1 ligand exerts its signaling effects by activating a heteromeric receptor of two transmembrane serine/threonine kinases, type I and type II receptors (TβRI and TβRII) [7,9]. TβRII transphosphorylates TβRI, activating its kinase function. Activated TβRI then phosphorylates the intracellular proteins Smad2 and Smad3. The phosphorylated Smad2 and Smad3 associate with Smad4, with the activated complex translocating to the nucleus where it interacts with other transcriptional co-activators and co-repressors to regulate expression of numerous genes [10]. This Smad-dependent signaling up-regulates expression of several transcription factors important for EMT induction, including Snail, Slug, Twist, and members of the ZFH family, ZEB1 (also called EF1, TCF8, AREB6, ZFHEP, NIL-2A, ZFHX1A, and BZP) and ZEB2 (also called SIP1, SMADIP1, ZFHX1B, and KIAA0569) [11-13].

Of particular importance are ZEB1 and ZEB2 because they are crucial regulators of EMT during embryonic development and cancer [14,15]. These transcription factors activate EMT by binding to E-box elements present in the E-cadherin promoter, suppressing synthesis of this cell-cell adhesion protein [16,17]. ZEB1 also promotes EMT by repressing expression of basement membrane components and cell polarity proteins [13,14,18,19]. ZEB2 has also been implicated in the induction of EMT [13]. The loss of E-cadherin and other epithelial structural components is a major event during EMT. Mutations in the *TCF8 *gene (GenBank accession number NM 030751) result in a mesenchymal to epithelial transition (MET) in mouse embryos by reprogramming gene expression, leading to developmental defects by diminishing progenitor cell proliferation and cell migration [20]. Thus, it is crucial to understand the role of ZEB1 and ZEB2 in the reversal of TGF-β-induced EMT.

Multiple signaling proteins in addition to Smads have been implicated in the induction of EMT by TGF-β1. These include Ras/MAPK [21], integrin β-1[22], integrin-linked kinase [23], p38 mitogen-activated protein kinase (p38 MAPK) [24], RhoA Kinase (ROCK) [25], phosphatidylinositol 3-OH kinase (PI3K) [26], Jagged1/Notch [27], SARA [28], nuclear factor kappa-B (NF-B) [29], Par6 [8,30], and ERK [31]. However, much less is known about how these signaling pathways and transcription factors maintain the mesenchymal program. Studies examining the reversal of EMT by perturbing one component of a signaling pathway with inhibitors or shRNAs demonstrate partial reversal of the mesenchymal state [32,33].

Here, we report full reversal of EMT morphology and patterns of gene expression by concurrently inhibiting TβRI kinase and ROCK. We show that inhibition of TβRI kinase blocks mesenchymal gene expression, an effect mediated by down-regulation of ZEB1 and ZEB2 levels, while the ROCK inhibitor stabilizes the epithelial structure. These findings demonstrate that combined use of TβRI kinase and ROCK inhibitors is important to decrease TGF-β signaling to enable full reversal of EMT.

## Results

### TGF-β1 induces EMT in mTEC-KO cells

**Figure 1:**
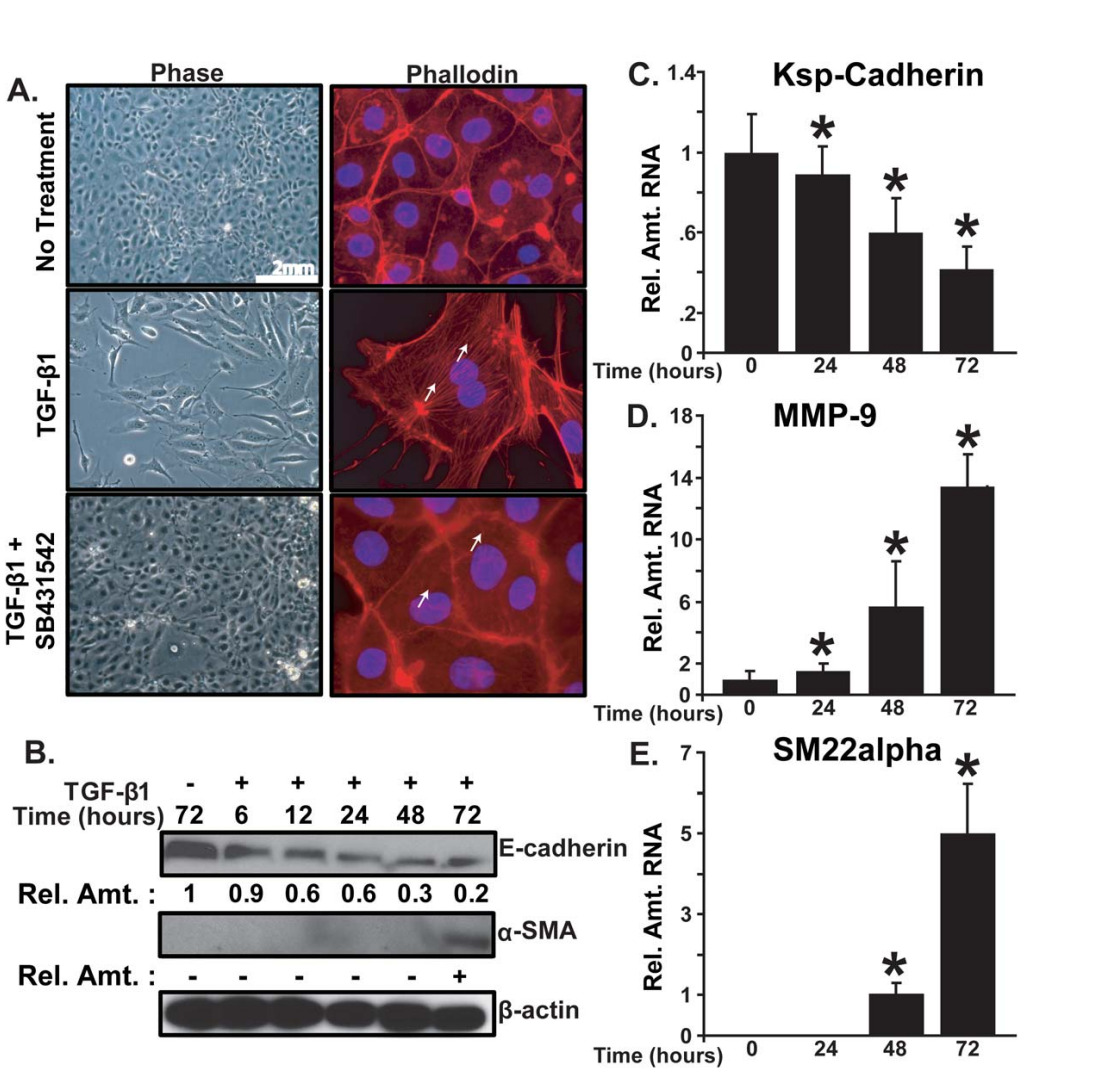
**TGF-β1 induces EMT in renal tubular epithelial cells**. **(A) **Phase contrast (left) and Phalloidin staining (right) of mTEC-KO cells incubated for 72 hours as indicated without TGF-β1, with 100 pM TGF-β1, or with TGF-β1 and 10 μM SB431542. Cell morphology was observed by bright field phase microscopy at 100× magnification. Phalloidin was used to detect F-actin at 400× magnification, while DAPI was used to detect nuclei. White arrows point to stress fibers. **(B) **Immunoblot showing protein expression of E-cadherin, α-SMA, and β-tubulin following incubation of mTEC-KO cells with 100 pM TGF-β1 for the indicated times. A dash indicates non-detectable levels of protein, while a plus sign indicates detectable protein levels over background. **(C-E) **show quantitative RT-PCR analysis of **(C) **Ksp-cadherin, **(D) **MMP-9, and **(E) **SM22 RNA levels in mTEC-KO cells incubated with TGF-β1 for the indicated times. Asterisks (*) indicate significant differences (*P < 0.05*, n = 9).

We used primary mouse tubular epithelial cells isolated from the renal cortex of TGF-β1 knockout mice (mTEC-KO cells) to model EMT in culture [34]. The mTEC-KO cells exhibit greater epithelial features than do wild-type renal epithelial cells. Renal tubular epithelial cells were chosen because of the correlation between the extent of tubulointerstitial fibrosis and the prognosis for end-stage renal disease [35]. In the absence of TGF-β1, mTEC-KO cells form an epithelial sheet; incubation with 100 pM TGF-β1 for 72 hours induced the mTEC-KO cells to acquire a more fibroblast-like, spindle shaped morphology indicative of mesenchymal cells (Figure 1A). Incubation with the TβRI inhibitor SB431542 blocked the TGF-β1-induced transition of the mTEC-KO epithelial cells into mesenchymal cells. The morphological transformation correlated with major changes in the actin cytoskeleton as revealed by phalloidin staining. Untreated epithelial cells exhibited a cortical actin staining below the cell membranes, whereas the TGF-β1-treated cells displayed elongated F-actin stress fibers. In the cells treated with the TβRI inhibitor SB431542, short, non-cortical actin fibers were detected.

The structural integrity and polarization of epithelial cells is maintained by E-cadherins binding to catenins and a network of actin filaments; reduction of E-cadherin expression is a hallmark of mesenchymal acquisition [36]. Thus, we also examined the expression levels of several genes regulated by TGF-β1 as markers for the epithelial and mesenchymal states. In mTEC-KO cells, incubation with TGF-β1 led to a significant decrease in expression of the epithelial protein E-cadherin and increase in expression of the mesenchymal protein α-smooth muscle actin (α-SMA) by 72 hours (Figure 1B).

Because TGF-β1 is known to regulate expression of multiple cadherins, we also examined expression of Kidney-specific cadherin (Ksp-cadherin). Ksp-cadherin has a similar developmental pattern of expression as the tight junction proteins ZO-1 and claudin-3 in kidney epithelial cells; therefore, it is used as a marker of the epithelial state [37,38]. Incubation with TGF-β1 led to a significant reduction in the level of Ksp-cadherin RNA (Figure 1C), while it led to significant increases in the RNA levels of mesenchymal markers matrix metalloproteinase-9 (MMP-9) (Figure 1D) and smooth muscle protein 22 (transgelin) (SM22) (Figure 1E). MMP-9 is an important extracellular matrix-degrading enzyme; SM22 has been shown to drive smooth muscle-specific gene expression *in vivo *[39-41]. Thus, we conclude that mTEC-KO cells completed the EMT program by several criterions following incubation with TGF-β1.

### A combination of TβRI inhibitor with either ROCK or p38 MAPK inhibitors is required for complete EMT reversal

**Figure 2:**
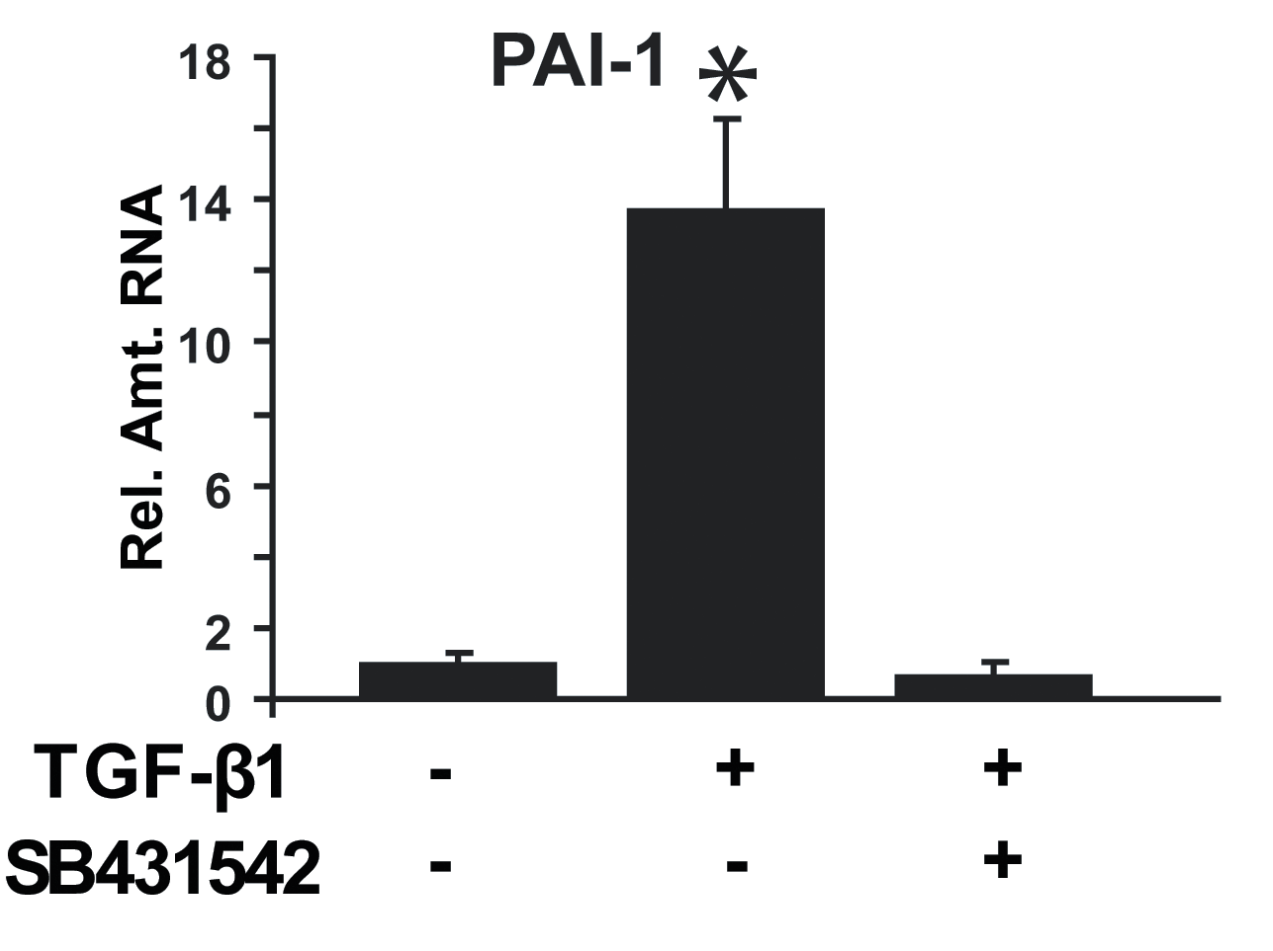
**Treatment with a TRβI inhibitor reverses PAI-1 RNA level in TGF-β1-induced mesenchymal renal tubular epithelial cells to levels present in epithelial cells**. mTEC-KOs were incubated with 100 pM TGF-β1 ligand for 72 hours, followed by the addition of 5 μM SB431542 plus 100 pM TGF-β1 for 24 hours. Cell lysates were prepared and relative PAI-1 RNA levels were determined by quantitative RT-PCR. Significant differences are indicated with an asterisk (*) (*P *<*0.05*, n = 9).

To examine the reversibility of EMT induced by TGF-β1 in mTEC-KO cells, we looked at the effects of five different kinase inhibitors targeting TβRI, p38 mitogen-activated protein kinase (p38 MAPK), MAP kinase kinase/extracellular signal-regulated kinase activator kinase (MEK1), c-Jun NH-terminal kinase (JNK), and Rho kinase (ROCK) with SB431542, SB203580, U0126, SP600125, and Y27632, respectively. These kinase inhibitors were previously implicated in EMT [24,31,42-44], 42-44 and their specificities have been well studied [45,46]. The cells were first incubated with 100 pM TGF-β1 for 72 hours to induce EMT, the kinase inhibitors were then added, and incubation was continued for an additional 24 hours. Addition of TβRI inhibitor SB431542 at 5 μM for 24 hours was sufficient to reduce significantly the RNA level of the TGF-β-responsive gene plasminogen activator inhibitor-1 (PAI-1) [47], demonstrating that TGF-β1 signaling was effectively inhibited (Figure 2).

**Figure 3:**
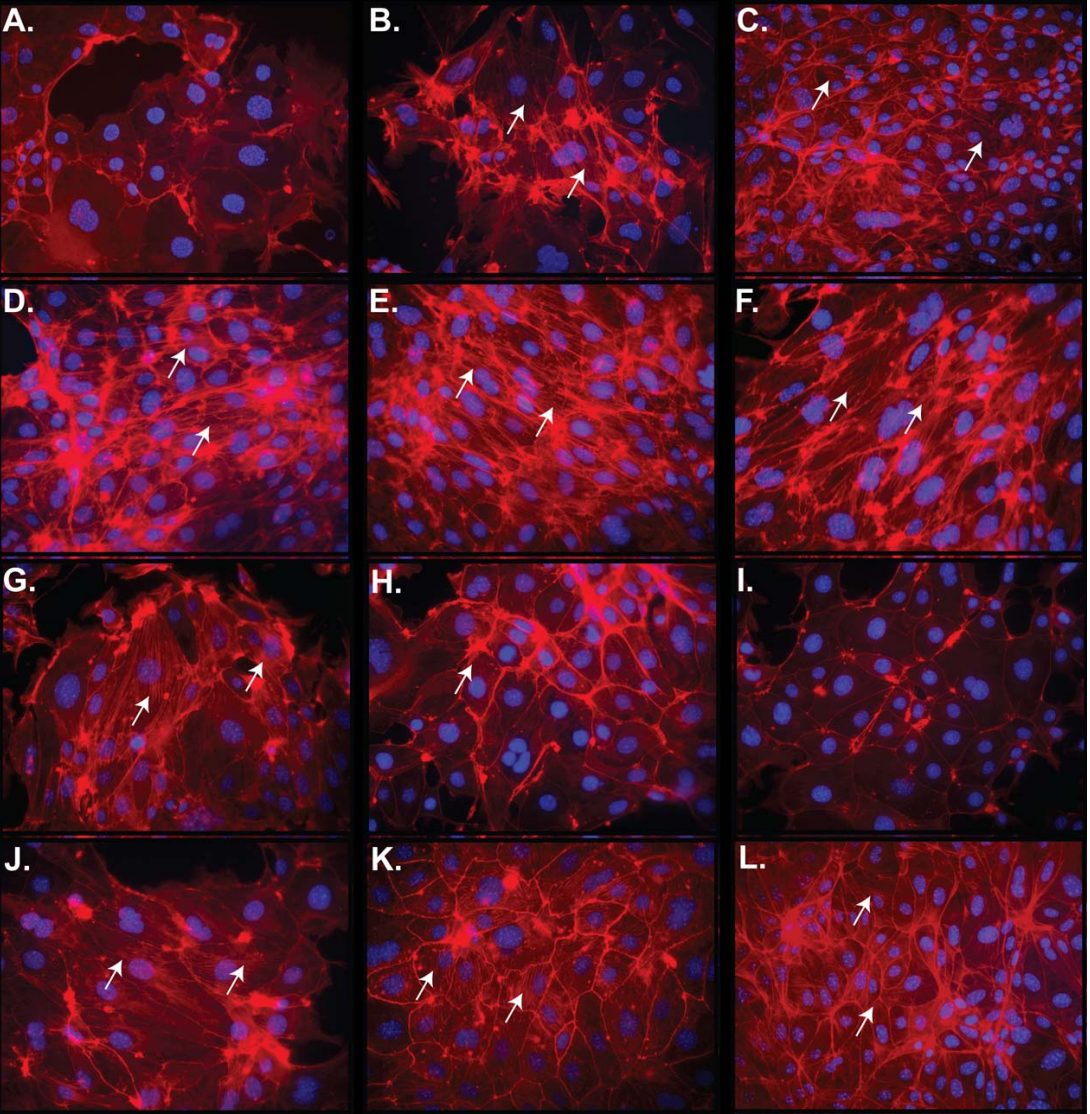
**Treatment of mTEC-KO cells with a TβRI inhibitor and a ROCK inhibitor together, but not individually, reverses the mesenchymal actin cytoskeleton stress fibers induced by TGF-β1**. mTEC-KO cells were incubated with 100 pM TGF-β1 for 72 hours, kinase inhibitors were added, and incubation was continued for an additional 24 hours. F-actin was visualized by staining with Texas Red-phalloidin. mTEC-KO cells were **(A) **untreated or treated with **(B) **100 pM TGF-β1 for 72 hours followed by **(C-G) **single kinase inhibitor or **(H-K) **SB431542 plus a second kinase inhibitor. Single kinase inhibitors and concentrations were as follows: **(C) **5 μM SB431542, **(D) **1 μM SB203580, **(E) **1 μM Y27632, **(F) **10 μM U0126, and **(G) **15 μM SP600125. Combinations of kinase inhibitors were 5 μM SB431542 with **(H) **1 μM SB203580, **(I) **1 μM Y27632, **(J) **10 μM U0126, or **(K) **15 μM SP600125. **(L) **Combination of 5 μM Y27632 and 5 μM SB203580. White arrows point to stress fibers.

To assess the effects of the kinase inhibitors on EMT, the actin cytoskeleton was examined by phalloidin staining. In contrast to its ability to prevent induction of EMT by TGF-β1 (Figure 1) and to reverse the elevation of PAI-1 expression (Figure 2), the TβRI inhibitor SB431542 failed to reverse the mesenchymal actin stress fiber morphology of the TGF-β1-treated mTEC-KO cells (Figure 3C). Inhibition of other kinases previously implicated in inducing EMT, such as p38 MAPK, MEK1, JNK, and ROCK, also did not reverse the actin stress fiber morphology induced in the mTEC-KO cells by TGF-β1 (Figures 3D-G). These results indicate that individual kinase inhibitors cannot fully reverse TGF-β1-induced EMT in mTEC-KO cells.

Since EMT effects are mediated by multiple cellular pathways, we also tested pair wise combinations of inhibitors of TβRI (5 μM SB431542), p38 MAPK (1 μM SB203580), ROCK (1 μM Y27632), MEK1 (10 μM U0126), and JNK (15 μM SP600125) (Figures 3H-K). We chose to use low doses of the inhibitors to reduce the chance of non-specific small molecule binding [45,48]. When the TβRI inhibitor SB431542 was combined with either p38 MAPK inhibitor SB203580 or ROCK inhibitor Y27632 for 24 hours, the epithelial appearance was restored (Figures 3H and 3I). The TβRI inhibitor SB431542 plus p38 MAPK inhibitor SB203580 (Figure 3H) reduced the presence of stress fibers more than either treatment by itself. However, non-cortical actin filaments were still detectable. Detectable actin stress fibers were eliminated by the combination of TβRI inhibitor SB431542 and ROCK inhibitor Y27632 (Figure 3I). Cortical actin bordering the cell-cell junctions was restored by both combinations. The addition of either MEK1 inhibitor U0126 or JNK inhibitor SP600125 along with TβRI inhibitor SB431542 had no detectable effect on the mesenchymal phenotype of the cells (Figures 3J and 3K). The combination of p38 MAPK inhibitor SB203580 and ROCK inhibitor Y27632 restored cortical actin staining, but stress fiber actin remained in the cells (Figure 3L). Increasing the concentration of TβRI inhibitor SB431542 to 10 μM led to a further decrease in the level of stress fibers; however, the combination of TβRI inhibitor SB431542 with a p38 MAPK inhibitor SB203580 or ROCK inhibitor Y27632 was more effective at eliminating them (Additional File 1). Similar results were observed in wild-type mTEC cells, with a combination of TβRI inhibitor SB431542 and ROCK inhibitor Y27632 reversing EMT as indicated by both gene expression (data not shown) and cell morphology (Additional File 2). Collectively, these data indicate that treatment of the cells with TβRI inhibitor SB431542 by itself cannot lead to full re-acquisition of cortical actin at the cell junctions.

**Figure 4:**
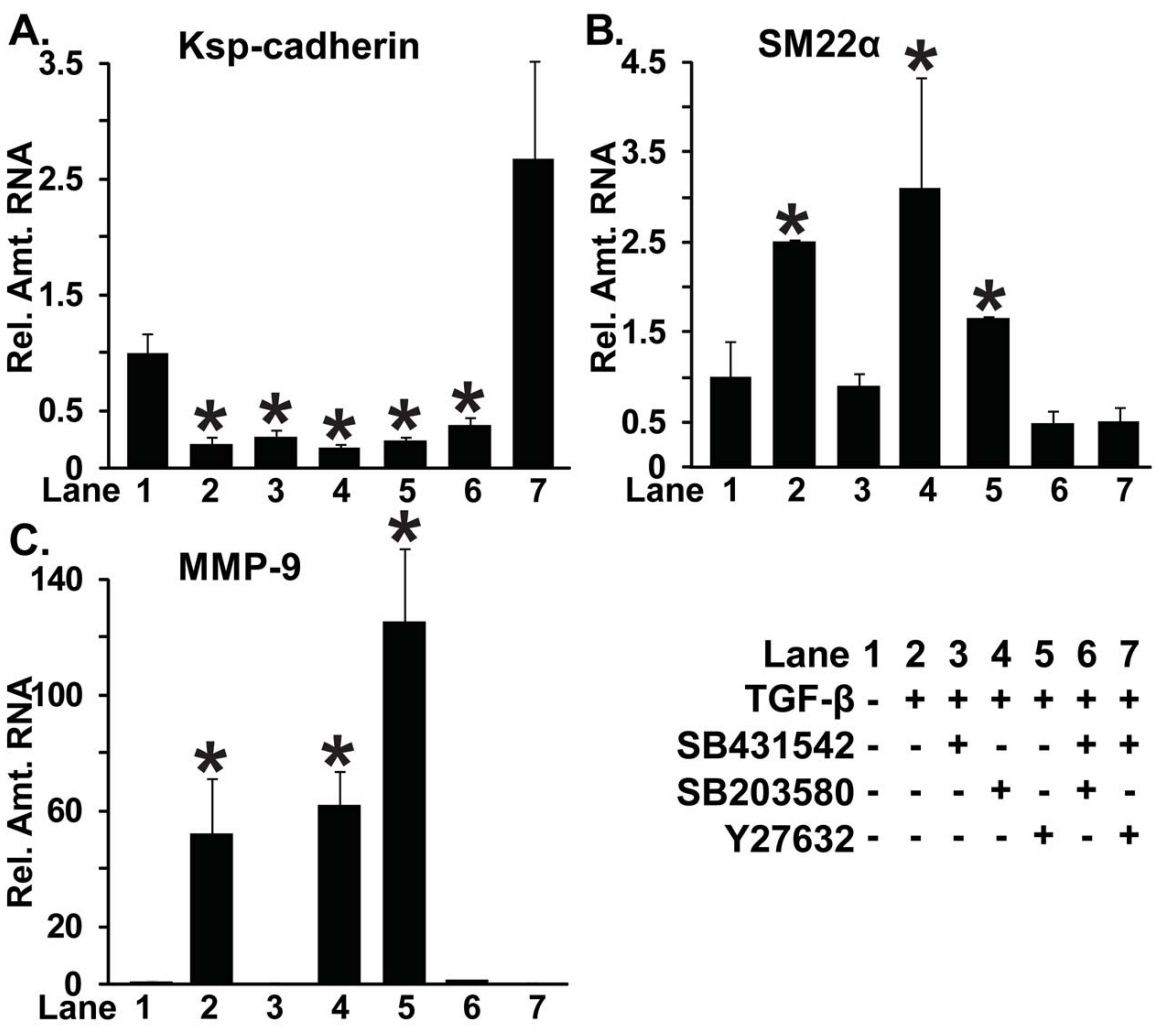
**Restoration of epithelial gene expression patterns requires a combination of kinase inhibitors**. mTEC-KO cells were incubated with 100 pM TGF-β1 for 72 hours to induce EMT. Afterward, they were incubated for an additional 24 hours with the indicated individual or combination of kinase inhibitors: 5 μM SB431542, 1 μM SB203580, and 1 μM Y27632. **(A) **Ksp-cadherin, **(B) **SM22, and **(C) **MMP-9 mRNA levels were determined by quantitative RT-PCR. Significant differences between the untreated cells **(lane 1) **versus cells treated with TGF-β1 **(lane 2) **or TGF-β1 followed by inhibitors **(lanes 3**-**7) **are indicated by asterisks (*) (*P < 0.05*, n = 9).

The effects of individual or combinations of kinase inhibitors on the expression of several genes altered by EMT were also examined by quantitative RT-PCR. The mTEC-KO cells were treated with 100 pM TGF-β1 to transition into the mesenchymal state; afterward, the kinase inhibitors were added. Incubation with TGF-β1 significantly reduced the Ksp-cadherin RNA level within 24 hours (Figures 1 and 4A). Addition of either TβRI inhibitor SB431542 or ROCK inhibitor Y27632 to the mesenchymal cells did not restore Ksp-cadherin RNA to pre-TGF-β1-levels (Figure 4A). Incubation with p38 MAPK inhibitor SB203580 led to a further decrease in Ksp-cadherin expression. The combination of TβRI inhibitor SB431542 plus p38 MAPK inhibitor SB203580 was not effective in increasing the Ksp-cadherin RNA level, but addition of TβRI inhibitor SB431542 together with ROCK inhibitor Y27632 led to a much greater increase in the Ksp-cadherin RNA level than the level achieved with either inhibitor by itself (Figure 4A). TβRI inhibitor SB431542 efficiently reduced SM22 and MMP-9 expression to pre-EMT levels (Figures 4B and 4C). The p38 MAPK inhibitor SB203580 did not reduce either the SM22 or MMP-9 expression level, indicating that presence of this p38 MAPK inhibitor failed to reverse expression of these genes associated with the mesenchymal state. The ROCK inhibitor Y27632 partially reduced SM22 expression (Figure 4B), but increased MMP-9 expression (Figure 4C). This increase in MMP-9 expression was prevented by treatment with TβRI inhibitor SB431542 combined with ROCK inhibitor Y27632 (Figure 4C). Thus, we conclude that the TβRI inhibitor SB431542 by itself is sufficient to induce the accumulation of some transcripts specific to epithelial cells; however, the combination of TβRI and ROCK inhibitors can effectively induce the accumulation of certain additional epithelial-specific transcripts such as Ksp-cadherin that correlate with the full reversal of EMT.

**Figure 5:**
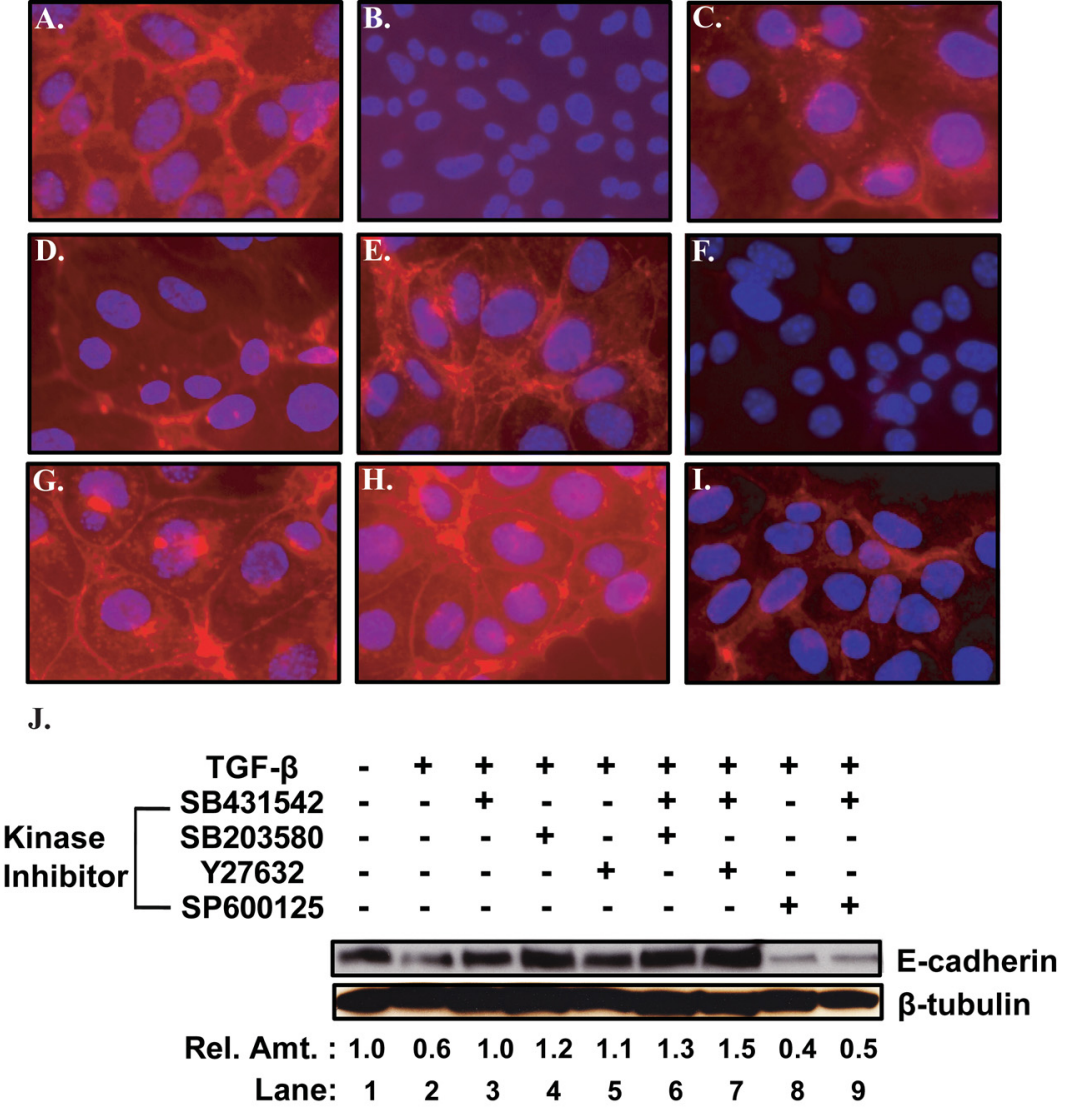
**E-cadherin expression is restored by combined treatment with a TβRI kinase inhibitor and either a ROCK or p38 MAPK inhibitor**. **A-I **Phase contrast microscopy of mTEC-KO cells incubated for 72 hours with 100 pM TGF-β1 followed by the indicated inhibitor(s) for an additional 36 hours prior to fixing and staining with DAPI. **(A) **untreated control cells; **(B) **treated only with TGF-β1; treated individually with the inhibitors **(C) **5 μM SB431542, **(D) **5 μM SB203580, **(E) **5 μM Y27632, or **(F) **15 μM SP600125; and treated together with 5 μM SB431542 plus **(G) **5 μM SB203580, **(H) **5 μM Y27632, or **(I) **15 μM SP600125. **(J) **Immunoblot analysis of cell lysates obtained from the above-treated mTEC-KOs for the presence of E-cadherin. β-tubulin served as a control.

One important criterion for epithelium restoration is re-expression of the cell-cell junction adhesion protein E-cadherin. To test for this factor, we incubated mTEC-KO cells with 100 pM TGF-β1 for 72 hours to induce EMT, added the indicated kinase inhibitors, and continued incubation for an additional 24 - 48 hours (Figure 5). Addition of the TβRI inhibitor SB431542 (Figure 5C), ROCK inhibitor Y27632 (Figure 5D), or p38 MAPK inhibitor SB203580 (Figure 5E) by itself led to partial reformation of E-cadherin at cell junctions compared to the TGF-β1-treated-mTEC-KOs (Figure 5B). Addition of the TβRI inhibitor SB431542 together with either p38 MAPK inhibitor SB203580 (Figure 5G) or ROCK inhibitor Y27632 (Figure 5H) restored E-cadherin localization to a level indistinguishable from that observed in the non-TGF-β1-treated cells (Figure 5A). JNK inhibitor SP600125 alone (Figure 5F) or a combination of TβRI inhibitor SB431542 plus JNK inhibitor SP600125 (Figure 5I) did not restore either the level or localization of E-cadherin. The combination of TβRI inhibitor SB431542 plus ROCK inhibitor Y27632 was most effective in restoring both localization of E-cadherin and its protein level as determined by immunoblot analysis of cell lysates (Figure 5J). Thus, we conclude that the TβRI, p38 MAPK, and ROCK inhibitors increase E-cadherin levels; however, the combination of the TβRI inhibitor with p38 MAPK or ROCK inhibitor is most effective.

### Reduction in ZEB1 levels is necessary for EMT reversal by TβRI inhibitor

**Figure 6:**
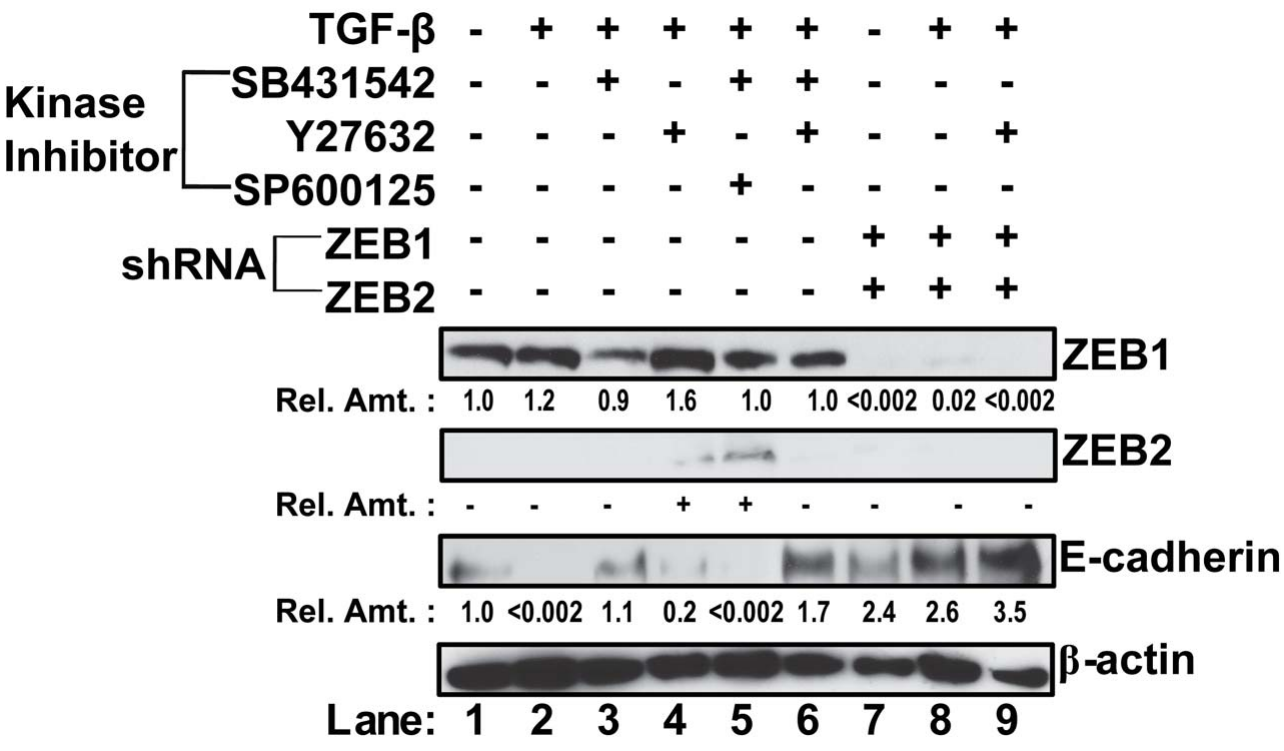
**Restoration of E-cadherin expression in NMuMG cells is dependent on levels of ZEB1 and ZEB2**. NMuMG cells were incubated for 48 hours with 100 pM TGF-β1 to induce EMT. Thereafter, the cells were incubated for an additional 24 hours with the indicated inhibitors: **(lane 3)**, 1 μM SB431542; **(lane 4)**, 1 μM Y27632; **(lane 5)**, 1 μM SB431542 and 10 μM SP600125; and **(lane 6)**, 1 μM SB431542 and 1 μM Y27632. NMuMG cells were also infected with shRNAmir-targeting ZEB1 and ZEB2 lentiviruses followed by incubation for an additional 24 hours without TGF-β1 **(lane 7)**, with 100 pM TGF-β1 **(lane 8)**, or with 100 pM TGF-β1 plus 1 μM Y27632 **(lane 9)**. Levels of ZEB1, ZEB2, E-cadherin, and β-actin protein in the cells were assessed by immunoblot analysis. Dash indicates protein present at level below background; plus sign indicates protein present at clearly detectable level. Similar findings were observed in two other independent experiments.

In the next series of experiments, we decided to examine the effects of ZEB1 and ZEB2 levels because their expression is regulated by TGF-β [13] (Additional File 3) and they are highly expressed in fetal kidney cells [49]. ZEB1 and ZEB2 can also play an important role in EMT induction by repressing E-cadherin expression [13,15,50-57]. Our data presented above led us to hypothesize that decreasing expression of transcriptional EMT regulators such as ZEB1 and ZEB2 is not sufficient for complete EMT reversal; rather, the presence of a ROCK inhibitor is also necessary to decrease mesenchymal structural components such as stress fibers. Historically, the effects of ZEB1 and ZEB2 have been studied in non-proximal tubule kidney cell lines such as Maderin Darby Canine Kidney (MDCK) cells [56,58]. We chose here to use Namru Murine Mammary gland (NMuMG) cells, a traditional EMT cell culture model [59], because: (i) NMuMG cells are easier to manipulate than mTEC-KO cells; (ii) they contain a readily detectable level of ZEB1 protein (Figure 6); (iii) we could only assay expression of ZEB1 and ZEB2 in mTEC-KO cells by quantitative RT-PCR (Additional File 3), not immunoblotting (data not shown); and (iv) RNA levels do not necessarily well reflect the protein levels of ZEB1 and ZEB2 ([60] A.L. Ellis & J.E. Mertz unpublished data) since ZEB1 and ZEB2 are highly regulated post-transcriptionally [55,56].

NMuMG cells were incubated with 100 pM TGF-β1 for 48 hours to induce EMT, the indicated kinase inhibitors were added, and incubation was continued for an additional 24 hours (Figure 6). Treatment of NMuMG cells with TGF-β1 led to a small increase in the level of ZEB1 protein (Figure 6, lane 2). Following incubation with TβRI inhibitor SB431542, the level of ZEB1 protein decreased back down to the level of untreated NMuMG cells (Figure 6, lane 3). Incubation with ROCK inhibitor Y27632 by itself led to a significant increase in the level of ZEB1 (Figure 6, lane 4); however, if cells treated with the ROCK inhibitor Y27632 were also incubated with TβRI inhibitor SB431542 (Figure 6, lane 6), the level of ZEB1 decreased to the level of untreated cells. ZEB2 protein was difficult to detect with our antibody; nevertheless, we could readily detect ZEB2 protein in the cells incubated with TβRI inhibitor SB431542 plus JNK inhibitor SP600125 (Figure 6, lane 5), indicating this combination of inhibitors led to increased expression of ZEB2 even if not ZEB1. From these results, we conclude that incubation with TβRI inhibitor can reverse the increase in ZEB1 levels.

We next tested whether the decrease in ZEB1 level by kinase inhibitors restored E-cadherin expression in NMuMG cells treated with TGF-β. Similar to our findings in the mTEC-KO model system, incubation with TGF-β1 led to loss of E-cadherin (Figure 6, lane 2). Incubation with either the TβRI inhibitor SB431542 (Figure 6, lane 3) or the TβRI inhibitor SB431542 in combination with ROCK inhibitor Y27632 (Figure 6, lane 6) restored the E-cadherin level. ROCK inhibitor Y27632 alone was not effective in restoring the E-cadherin level (Figure 6, lane 4). E-cadherin was also not restored in cells incubated with TβRI inhibitor SB431542 plus JNK inhibitor SP600125 (Figure 6, lane 5). Although the ZEB1 level was similar to the cells incubated with the TβRI inhibitor SB431542 and ROCK inhibitor Y27632 (Figure 6, lane 6), the cells incubated with TβRI inhibitor SB431542 plus JNK inhibitor SP600125 also expressed ZEB2 (Figure 6, lane 5) which could account for the observed repression of E-cadherin expression. These data indicate that inhibition of the TGF-β-induced increase in ZEB1 levels can lead to re-expression of E-cadherin. However, the re-expression of E-cadherin can be inhibited if ZEB2 is expressed.

To test whether ZEB1 and ZEB2 levels directly affect E-cadherin expression, we performed RNA-mediated interference experiments. NMuMG cells infected with lentiviruses expressing a pool of individual ZEB1 and ZEB2 shRNAs knocked down endogenous expression of ZEB1 to a nearly undetectable level within 72 hours regardless of whether the cells had been treated with TGF-β1 (Figure 6, lanes 7-9). Although ZEB2 protein was not detected by our assay in these cells, we included shRNAs targeting ZEB2 because others reported detection of ZEB2 RNA in TGF-β1 treated NMuMg cells [13]. While incubation with TGF-β1 led to loss of E-cadherin (Figure 6, lane 2), this treatment with ZEB1 plus ZEB2 shRNAs restored E-cadherin to levels that were higher as compared to the original cells (Figure 6, lane 8). ZEB depletion together with incubation with 1 μM Y27632 also led to increased E-cadherin expression (Figure 6, lane 9). Thus, we conclude that depletion of ZEB by either shRNAs or kinase inhibitors is sufficient to re-introduce E-cadherin expression in TGF-β-induced mesenchymal cells.

### ZEB1 depletion combined with ROCK inhibitor Y27632 is required to complete the EMT reversal program by eliminating stress fibers

**Figure 7:**
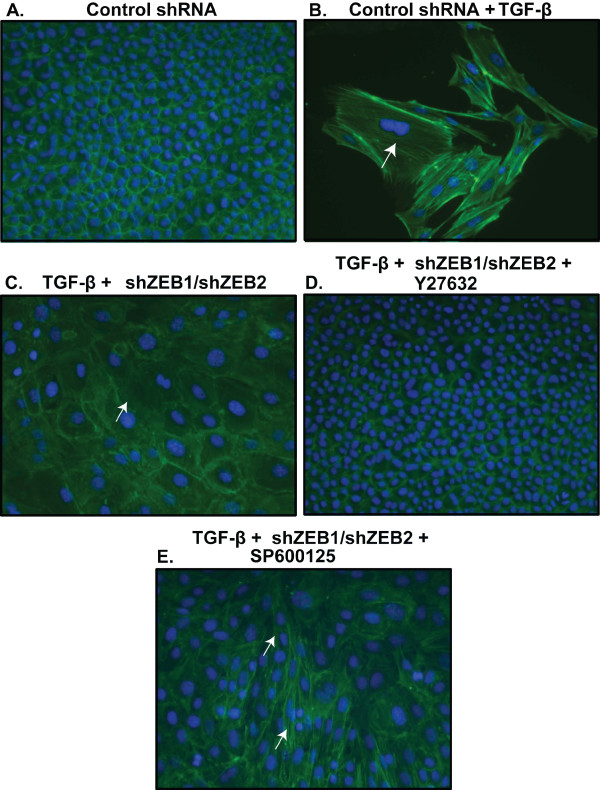
**ZEB1 and ZEB2 depletion by shRNAs in NMuMG cells attenuates F-actin stress fibers**. Cells were treated as indicated with 100 pM TGF-β1 for 48 hours followed by an additional 72 hour incubation with a lentivirus encoding pLKO.1 (a control shRNA) or lentiviruses encoding shRNAs against ZEB1 and ZEB2, with 1 μM Y27632 or 10 μM SP600125 added during the last 24 hour incubation with the shRNAs. F-actin was visualized by phalloidin. Cells were viewed at a 400× magnification. White arrows point to stress fibers.

Loss of E-cadherin is accompanied by rearrangement of the actin cytoskeleton to maintain polarized cell structure. NMuMG cells treated with TGF-β exhibit stress fibers and lower cell number (Figure 7B). Thus, we also examined the effect of ZEB level on the arrangement of F-actin stress fibers in NMuMG cells. Treatment of the cells with shRNAs against ZEB1 and ZEB2 led to attenuation of the stress fibers (Figure 7C); however, the arrangement of F-actin did not completely reverse as compared to the cells incubated with the kinase inhibitors (Figure 3). On the other hand, NMuMG cells treated with TGF-β and incubated with ROCK inhibitor Y27632 together with the ZEB shRNAs exhibited decreased F-actin fibers and reappearance of cortical actin (Figure 7D). This failed to occur when TGF-β-treated cells infected with the viruses expressing the shRNAs against the ZEBs were incubated with JNK inhibitor SP600125 (Figure 7E). Taken together, these data indicate that ROCK inhibitor Y27632 treatment leads to stabilization of cortical actin, while reduction in expression of the ZEBs leads to increased expression of factors such as E-cadherin necessary for EMT reversal.

## Discussion

**Figure 8:**
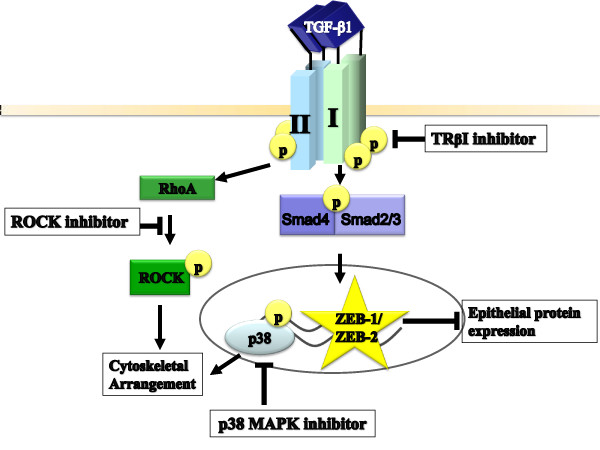
**Model for reversal of EMT induced by TGF-β1**. To re-express epithelial proteins such as E-cadherin, a TβRI kinase inhibitor is needed to decrease expression of mesenchymal genes (*e.g*., ZEB1 and ZEB2), while a Rho kinase inhibitor is required to stabilize the epithelial cortical actin. A p38 MAPK inhibitor may also be useful as this drug in conjunction with the TβRI kinase inhibitor can further EMT reversal by reducing stress fiber actin formation.

The goal of this study was to elucidate molecular mechanisms involved in maintaining the mesenchymal state induced by TGF-β1. Here, we demonstrated that preventing EMT requires blocking the TβRI kinase (Figure 1), while reversing the EMT program is more complex, requiring inhibition of both TβRI kinase and ROCK (Figures 3, 4, 5, 6). A p38 MAPK inhibitor also plays a role by working in conjunction with the TβRI kinase inhibitor to further lessen the mesenchymal structural elements to reverse EMT (Figure 3). We defined the success of an agent in reversing EMT as the re-expression of key epithelial proteins (*e.g*., E-cadherin, cortical actin) and the re-positioning of these proteins to allow for epithelial cell morphology. We also demonstrated that reversal of EMT by the TβRI inhibitor SB431542 involves, in part, inhibiting expression of ZEB1, a key transcriptional repressor of E-cadherin expression and the epithelial state (Figure 6). Taken together, these findings indicate that TGF-β maintains the mesenchymal phenotype through sustained activation of Smad-dependent transcriptional responses and elements downstream from ROCK (Figure 8).

### Decreasing ZEB1 and ZEB2 expression enables partial re-programming of EMT by inducing E-cadherin expression

The levels of ZEB1 and ZEB2 can regulate the epithelial transition to the mesenchymal state, [51-56,60,61]. In Madin-Darby Canine Kidney (MDCK) cells, EMT is preceded by the loss of mature miR200a-c, inducing up-regulation of ZEB1 and ZEB2 expression followed by loss of E-cadherin expression and transition to the mesenchymal state [50,59,62,63]. In cancers or fibrosis, this feedback loop may be disrupted, leading to unregulated expression of ZEB1 or ZEB2. To regain this feedback loop, a small molecule inhibitor of TβRI may be useful to block factors maintaining the mesenchymal state. In this case, blocking the feedback loop during TGF-β1-induced maintenance of ZEB1 levels might enable re-expression of miRNA200 family members and proteins involved in epithelial cell morphology (Figure 8).

Thuault *et al*. [33] previously examined the role of Snail, another E-box-binding transcriptional repressor of E-cadherin gene expression, in EMT reversal. They reported that High Mobility Group A2 (HMGA2) sustains TGF-β-induced EMT in NMuMG cells, with partial EMT reversal occurring as measured by restoration of tight junction proteins and a partial restoration of cortical actin when Snail is targeted for depletion by shRNA. However, E-cadherin did not re-colocalize or become expressed at cell-cell junctions, indicating that either Snail was not sufficiently knocked down in their experiment or there was another factor regulating localization of the cytoskeleton components.

Our studies demonstrate that depleting mesenchymal cells of ZEB1 and ZEB2 with specific shRNAs or using a TRβI inhibitor in combination with a ROCK inhibitor is sufficient to restore fully E-cadherin protein levels (Figures 6 and 7). However, solely targeting ZEB1 and ZEB2 with shRNAs did not lead to full restoration of cortical actin at the cell borders; rather, treatment as well with a ROCK inhibitor was necessary for complete reduction of stress fibers (Figure 7). Other factors may also be necessary to maintain the epithelial cytoskeleton.

### ROCK regulates the cytoskeleton during EMT reversal to stabilize the epithelial structure

One plausible regulator of the actin cytoskeleton is Rho. Here, we showed that ROCK is responsible for only a subset of EMT changes, such as actin rearrangement (Figures 3 and 7). Inhibiting ROCK was not sufficient to induce E-cadherin or other epithelial characteristics (Figures 3, 4, 5, 6). This finding implies that ROCK is necessary for epithelial cells to regain cytoskeletal structure. We hypothesize that re-acquisition of the epithelial cytoskeleton might sequester the mesenchymal signaling associated with the unformed cell-cell adhesions [64]. In mammary gland epithelial cells, Rho location is controlled by the partitioning-defective protein 6C (Par6), a regulator of the polarity complex [8]. When TβRII is activated, Par6 is phosphorylated and recruits the E3 ubiquitin ligase Smurf1 to the cell membrane, thereby regulating the localization of Rho by ubiquitination. This implies that the location of Rho is important for the arrangement of actin in epithelial cells. To determine the mechanism of TGF-β activation of stress fibers, further studies are needed to examine if TGF-β induces F-actin stress fibers as the result of ROCK activating LIM kinase and cofilin [65,66] or by ROCK regulating gene expression through Jak-Stat and NF-B pathways [67].

### Temporal control of EMT reversal varies with the agents and cell type

Other reports of mesenchymal phenotypic reversion utilizing inhibitors have claimed various degrees of success. For example, EMT induced in EpH4 mouse mammary epithelial cells by an estradiol-inducible c-Fos-estrogen receptor fusion protein was only partially reversed after 3 - 6 days of incubation with BIBU 3029, a small molecule inhibitor of TβRI kinase [68]. However, ectopic expression of E-cadherin combined with addition of BIBU 3029 did lead to full reversal of the EpH4 mesenchymal cells as assayed by the formation of cobblestone-like epithelial sheets with tight junctions between the cells and localized expression of E-cadherin and β-catenin at cell junctions, but only after 6 days. Others have reported that incubation with individual inhibitors of TβRI kinase is sufficient to increase E-cadherin expression and to induce a more epithelial morphological appearance within 48 hours in several cell lines [69-71]. By contrast, our study showed that a combination of a TβRI inhibitor and a ROCK inhibitor can enable complete, rapid reversal of EMT within 24 hours, including re-expression of Ksp-cadherin and E-cadherin (Figures 4 and 5). Plausible explanations for the differences in our observations include (i) the agents employed to induce EMT, and (ii) the specific cell types used in the experiments.

### Chemical inhibition of JNK blocks EMT reversal by the TβRI inhibitor

Our studies demonstrate that small molecule inhibition of JNK can block the reversal effects of the TβRI inhibitor by maintaining stress fibers and decreasing E-cadherin levels (Figures 3, 5, 6, 7). Suppression of JNK leads to increased expression of the transcription factor Slug in trophoblast stem cells, leading to induction of an EMT state [72]. Like ZEB1 and ZEB2, Slug induces EMT by repressing expression of E-cadherin via binding to E-box elements in the E-cadherin promoter [73]. Another plausible explanation for maintenance of non-TGF-β-dependent EMT is that the JNK inhibitor may activate other pathways such as NF-B [29]. Previous studies demonstrated NF-B both suppresses apoptosis and induces EMT in breast cancer cells [74]. NF-B has been shown to induce EMT by upregulating E-cadherin transcriptional repressors such as Snail, Slug, ZEB1, and ZEB2 [29]. This indicates that a JNK inhibitor should not be used in conjunction with a TβRI inhibitor as doing so may compromise EMT reversal.

### Inhibitors in combination may be a feasible therapeutic approach for treating patients with EMT-associated diseases

In chronic fibrotic diseases, reversal of the mesenchymal state generated by EMT may be critical for restoring function to organs. For example, it might provide a potential therapy for treating chronic kidney damage caused by constitutively high levels of TGF-β1 [3]. Blocking EMT is useful for preventative medicine. However, reversing EMT holds more promise for treating existing diseases. Our use of small molecule inhibitors of individual protein kinases not only demonstrates their potential for dissecting mechanisms of signal transduction for specific ligands and for delineating their roles in biologic responses, but also their potential as therapeutic agents. Yingling *et al*. have described a group of competitive ATP-binding site inhibitors of ALK-5 as possible agents for treating some cancers and fibrosis [75]. In certain cancers, treatment with a TβRI inhibitor shows promise in halting metastasis [33,68-70,76,77]. Therapeutics in the form of growth factors such as BMP-7 can also act as TGF-β antagonists to treat fibrotic disease [78]. Other approaches to block TGF-β activity, such as anti-sense DNA targeting TGF-β, are in clinical trials as a vaccine against tumor cells or as therapeutics for treating patients with high-grade gliomas [79,80]. In addition, ROCK and p38 MAPK inhibitors are in clinical trials as potential therapeutics targeting a variety of cancers [81,82]. These recent developments suggest multiple therapeutic strategies may be possible for treating patients with diseases in which TGF-β-induced EMT contributes to the pathology.

## Conclusion

We showed here that reversing EMT in mTEC-KO cells requires inhibition of both TβRI kinase and ROCK. The TβRI kinase inhibitor decreased expression of ZEB1 and ZEB2, thereby increasing expression of the epithelial protein E-cadherin, and the ROCK inhibitor was necessary to fully eliminate mesenchymal actin stress fibers (Figure 8).

## Methods

### Cells and Reagents

Early passage (passage 2) TGF-β1 knockout murine renal tubular epithelial cells (mTEC-KO) and early passage (passage 5) murine renal tubular epithelial cells (mTEC-WT) were generously provided to us by Dr. Jeffrey Kopp (National Institute of Diabetes and Digestive and Kidney Diseases, Bethesda, MD). The cells were grown until passage 20. They were maintained in Renal Epithelial Cell Growth Medium (Cambrex, MD) supplemented with 0.25% fetal bovine serum (FBS), a Bullet Kit that contained epidermal growth factor, insulin, hydrocortisone, GA-1000, epinephrine, T_3_, and transferrin (Cambrex, NJ), and penicillin and streptomycin (Gibco-Invitrogen, CA). Namru murine mammary gland (NMuMG) cells were obtained from Dr. Caroline Alexander (UW-Madison, WI). They were grown in DMEM supplemented with 10% FBS, 10 μg/ml insulin, 100 μg/ml penicillin, and 100 U/ml streptomycin. 293T cells were purchased from ATCC (VA). They were grown in 10% DMEM supplemented with 10% FBS, 100 μg/ml penicillin, and 100 U/ml streptomycin. All cells were maintained in a 37°C humidified 5% CO_2 _incubator. Carrier-free TGF-β1 was obtained from R&D Systems (MN). Chemical inhibitors SB203580, SP600125, and Y27632 (Calbiochem, CA); SB431542 (Sigma, MO); and U0126 (Promega, WI) were aliquoted after reconstitution and frozen at -80°C.

### Production of Lentiviruses

The lentiviral shRNAmir vectors targeting ZEB1 (target set NM_011546), ZEB2 (target set NM_014795), and control pLKO.1 (RHS4080) were purchased from OpenBiosystems (Thermo Scientific, AL). Plasmid pLKO.1 contains a scrambled sequence that results in synthesis of a shRNA that does not appear to inhibit expression of any known cellular gene. To generate virus, mycoplasma-free 293T cells were transfected using LT1 (Mirus, WI) with 10 μg total of a mixture containing shRNAmir ZEB1 and ZEB2 or pLKO.1 as indicated, 3 μg lentiviral DNA encoding Gag/Pol, and 1 μg VSVG [83] (provided by Dr. Bill Sugden, Madison, WI). The medium was supplemented with 50 mM HEPES solution, pH 7.3 (Invitrogen) and changed after 6 hours. Viral supernatant was collected after 24 hours and passed through a 0.45 μm filter. NMuMG cells were infected daily with the pool of shZEB1 and shZEB2 viruses over 48 - 72 hours to decrease ZEB1 and ZEB2 expression.

### Immunoblotting

After treatment as indicated, cells were washed with cold PBS, lyzed in TNE buffer [50 mM Tris-HCl (pH 8.0), 1% NP40, 150 mM NaCl, 5 mM EDTA] and pelleted by centrifugation at 14,000 rpm for 5 min at 4°C. Lysates were prepared using TNE buffer supplemented with protease inhibitor cocktail (Roche, NJ) and protease inhibitor cocktail solution III (Calbiochem, CA). Cell homogenates were incubated for 10 min at 100°C in 2× loading buffer. Equal amounts of protein, as assessed by BCA Protein Assay Kit (BioRad, CA), were added to each well. The proteins were separated by electrophoresis in 4% - 20% gradient polyacrylamide gels (ISC BioExpress, UT) and transferred to PVDF membranes (Millipore, MA) or nitrocellulose filters (ISC Biosystem, UT). The primary antibodies used for detection were as follows: E-cadherin (BD Biosciences, CA), ZEB1 (H102, Santa Cruz Biotechnology, CA), ZEB2 (Dr. Michel Sanders, University of Minnesota, MN; purified by Dr. Xianming Yu, UW-Madison, WI), and α-Smooth Muscle Actin (Sigma). Anti-mouse IgG conjugated with horseradish peroxidase (Millipore) was used as the secondary antibody. Blots were developed by ECL (GE Healthcare, NJ). Where indicated, the immunoblots were stripped by incubation with 100 mM β-mercaptoethanol, 2% SDS, 62.5 mM TRIS (pH 8.2) at 65°C for 1 hour and reprobed with β-actin (Sigma) or β-tubulin (Sigma) primary antibody as indicated and HRP secondary antibody (GE Healthcare). Relative protein levels were determined by densitometry using Bio-Rad Quantity One Software (Bio-Rad), with normalization to the amount of cellular β-actin or β-tubulin present in each sample. Changes in the amount of a protein present in an experimental sample are shown relative to the amount of this protein present in the untreated control sample processed in parallel.

### Quantitative RT-PCR

After treatment as indicated, total RNA was isolated from the cells using RNAeasy Miniprep kit (Qiagen) and quantified by UV spectrophotometer. 1.5 μg of RNA from each sample was converted by reverse transcriptase into cDNA using an OmniScript kit (Qiagen). Primers used for qRT-PCR were as follows: mouse Ksp-cadherin: forward-5'-CTGCACACAGAAGTCCCTGA-3', reverse 5'-CCTTGTCGCCACTAGAAAGC-3'; mouse MMP-9: SuperArray primer (MD) PPM03661A; mouse SM22: forward 5'-GCAGTCCAAAATTGAGAAGA-3', reverse 5'-CTGTTGCTGCCCATTTGAAG3'; mouse PAI-1: forward 5'-TTCAGCCCTTGCTTGCCTC-3', reverse 5'-ACACTTTTACTCCGAAGTCGGT-3'; ZEB1: forward 5'-AACGGAGATTTGTCTCCCAGT-3', reverse 5'-CTGTCCAGCTTGCATCTTTTC-3'; ZEB2: forward 5'-TAGCCGGTCCAGAAGAAATG-3', reverse 5'-GGCCATCTCTTTCCTCCAGT-3'[20]; mouse GAPDH: forward 5^'^-AGGTCGGTGTGAACGGATTTG-3^'^, reverse 5^'^-TGTAGACCATGTAGTTGAGGTCA-3^'^; and P0: forward, 5'-GACAATGGCAGCATCTACAAC-3', reverse, 5'-GCAGACAGACACTGGCAAC-3' [84]. cDNA was amplified in an Opticon 2 PCR machine (MJ Research) and labelled using an ABI SYBR Green qPCR Kit (ABI Systems, CA). The amplifications were carried out in the following manner: initial denaturation for 10 min at 95C, denaturation for 10 sec at 95°C, annealing for 30 sec at an optimal temperature for each cDNA, and extension for 30 sec at 72°C. Standards were created from Pfu (Stratagene, CA)-amplified PCR products purified by gel electrophoresis. Glyceraldehyde-3-phosphate dehydrogenase (GAPDH) was used as an internal control to normalize gene expression levels, except for ZEB1 and ZEB2 where the ribosomal protein P0 was used, instead. Relative ZEB1 and ZEB2 RNA levels were calculated by a modified Ct method [85].

### Immunofluorescence

Cells were seeded on glass cover slips (Fisher, PA) or 8-well chamber slides (Fisher) and cultured as described. Afterward, the cells were fixed by incubation in 4% paraformaldehyde for 5 min and permeabilized in PBS containing 0.1% Triton X-100 for 10 min. Non-specific binding was blocked with 10% BSA or normal goat serum for 30 min. F-actin was stained at a 1:1000 dilution in 2% BSA of Texas Red-conjugated or FITC-conjugated phalloidin (Sigma) for at least 30 min. E-cadherin (BD Biosciences) was diluted 1:50 in 2% normal goat serum and incubated overnight. A secondary antibody made in goat (Invitrogen) was used at a 1:1000 dilution and incubated for 2 hours in a humid chamber with minimal exposure to light. All washes were done in 1× PBS. An anti-fade solution containing DAPI (Vector Laboratories, CA) was used in mounting the slides. Images were taken at room temperature with an Axiocam digital camera attached to a Zeiss microscope. Axiovision was used to acquire the image. Adobe Photoshop was used to merge images.

### Statistical Analysis

Unless indicated otherwise, all experiments were performed on three separate occasions, each time with triplicates, with the figures showing means of the triplicates for one of the experiments (n = 3). For statistical evaluation, the data from all experimental replicates were pooled (n = 9). For comparisons between pairs, we used one-way analysis of Wilcoxson Rank Sums determined with MSTAT software http://mcardle.oncology.wisc.edu/mstat. A *p *value < 0.05 was considered statistically significant.

## Abbreviations

EMT: epithelial to mesenchymal transition; Ksp-cadherin: kidney specific cadherin; JNK: c-Jun NH-terminal kinase; MEK1: MAPK/extracellular signal-regulated kinase; MMP-9: matrix metalloprotease-9; mTEC-KO: murine tubular epithelial cells from TGF-β knockout mouse; p38 MAPK: p38 mitogen-activated protein kinase; NMuMG: Namru murine mammary gland; ROCK: Rho kinase; SM22: smooth muscle protein 22; α-SMA: alpha smooth muscle actin; TGF-β: Transforming Growth Factor β; TβRI: Transforming Growth Factor-β Receptor Type I.

## Authors' contributions

SD and FMH designed and planned the experiments and analyzed the data for the inhibitor studies. SD and JEM designed and planned the experiments for the ZEB studies. SD carried out all of the experimental work, analyzed the data, and prepared the manuscript. FMH and JEM edited the manuscript. All authors read and approved the final manuscript. FMH and JEM supervised the overall conduct of the research and provided funding. BNB provided mTEC-KO cells, mTEC-WT cells, expertise on kidney fibrosis, and funding.

## Supplementary Material

Additional File 1 **Higher dose of kinase inhibitors by themselves does not reverse stress fiber actin in mTEC-KO cells; rather, a combination of TβRI inhibitor and a ROCK inhibitor is required to reverse EMT**. mTEC-KO cells were incubated with 100 pM TGF-β1 for 72 hours, kinase inhibitors were added, and incubation was continued for an additional 24 hours. F-actin was visualized by staining with Texas Red-phalloidin. Cells were viewed with an oil-objective lens at a 630× magnification. mTEC-KO cells were **(A) **untreated or treated with **(B) **100 pM TGF-β1 for 72 hours followed by **(C-E) **single kinase inhibitor or **(F-G) **SB431542 plus a second kinase inhibitor. Single kinase inhibitors and concentrations were as follows: **(C) **10 μM SB431542, **(D) **10 μM SB203580, and **(E) **10 μM Y27632. Combinations of kinase inhibitors were 10 μM SB431542 with **(F) **10 μM SB203580 and **(G) **10 μM Y27632. White arrows point to stress fibers. Click here for file

Additional File 2 **A combination of TβRI inhibitor and a ROCK inhibitor is required to reverse EMT in mTEC-WT cells**. mTEC-WT cells were incubated with 100 pM TGF-β1 for 72 hours, kinase inhibitors were added, and incubation was continued for an additional 24 hours. F-actin was visualized by staining with Texas Red-phalloidin. mTEC-WT cells were **(A) **untreated or treated with **(B) **100 pM TGF-β1 followed by **(C) **10 μM SB431542 plus 10 μM Y27632. White arrows point to stress fibers. Click here for file

Additional File 3 **TGF-β1 induces ZEB1 and ZEB2 RNA accumulation in mTEC-KO cells**. mTEC-KO cells were incubated for the times indicated with 100 pM TGF-β1. Cells were harvested and assayed by quantitative RT-PCR for ZEB1 and ZEB2 RNA. Data shown are means + S.E.M.s of two experiments performed in triplicate. Asterisk (*) indicates significant difference (*P < 0.05*, n = 6). Click here for file
