# Supplementary figures and images for: Complete reversal of epithelial to mesenchymal transition requires inhibition of both ZEB expression and the Rho pathway
Source: BMC Cell Biol. 2009 Dec 21;10:94. doi: 10.1186/1471-2121-10-94 (PMC2806300; doi:10.1186/1471-2121-10-94)

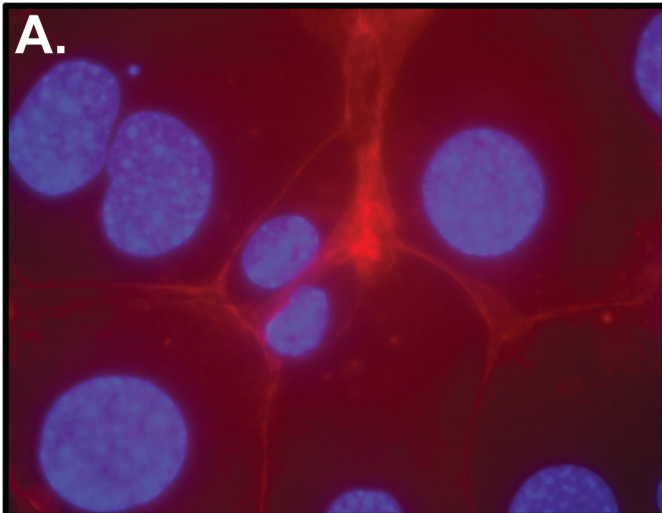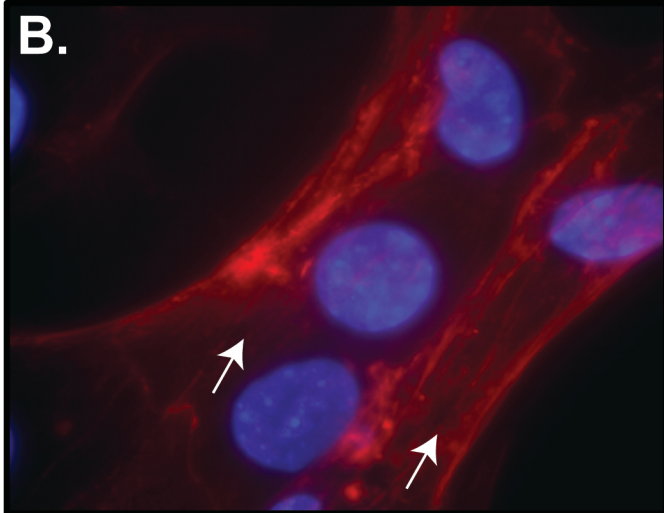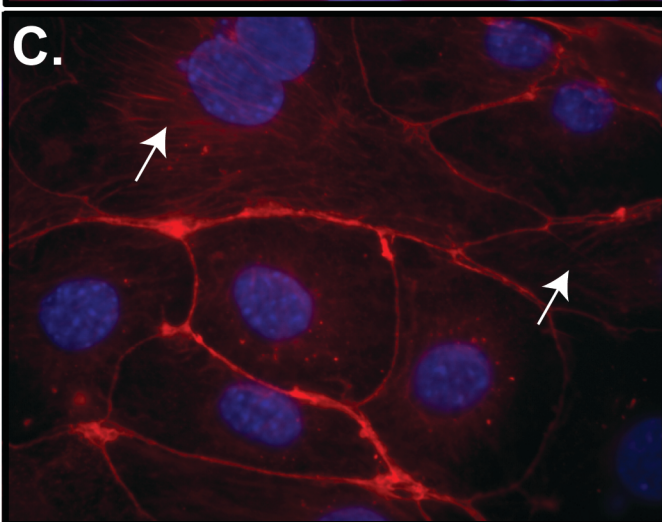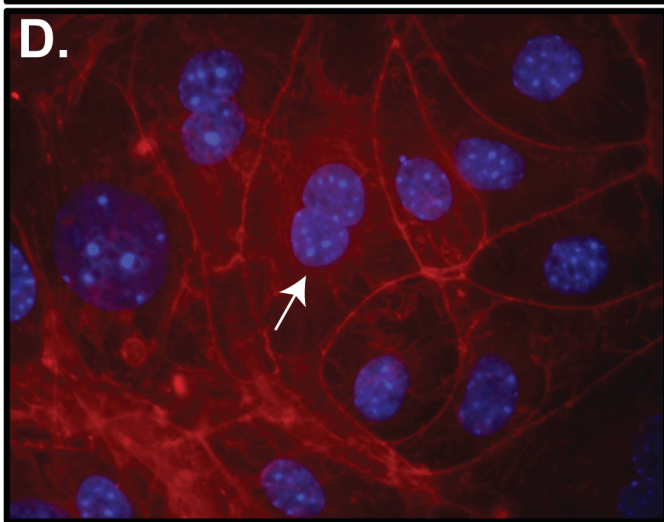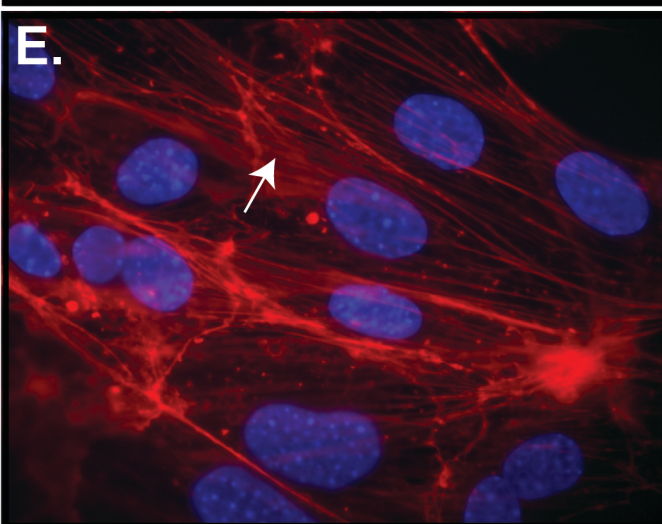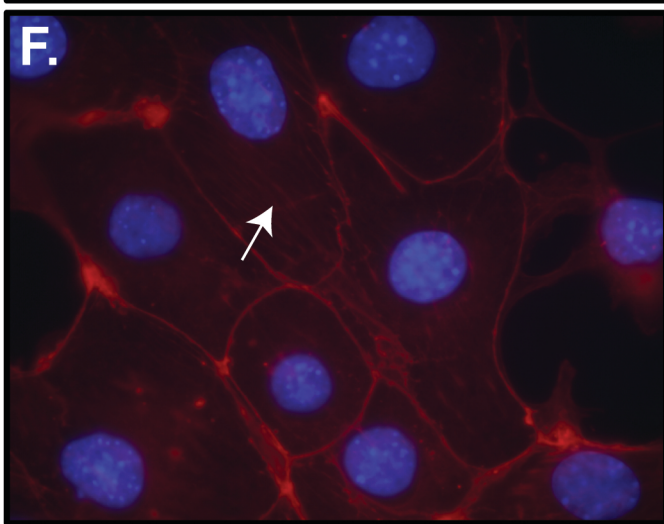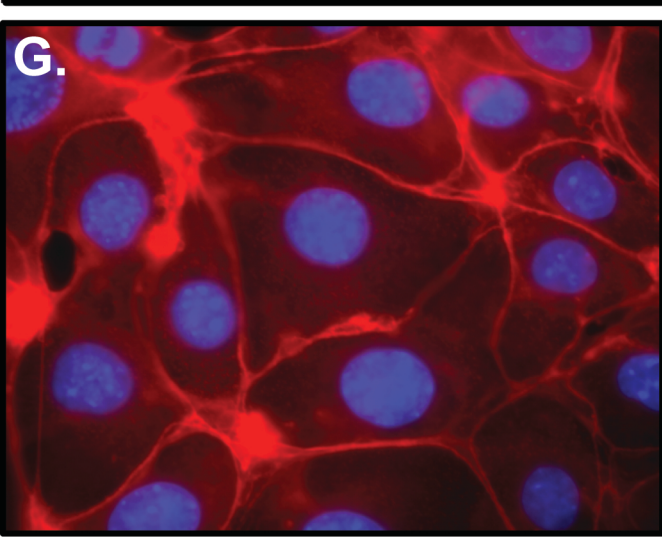

Supplement: Additional File 1 — Higher dose of kinase inhibitors by themselves does not reverse stress fiber actin in mTEC-KO cells; rather, a combination of TβRI inhibitor and a ROCK inhibitor is required to reverse EMT. mTEC-KO cells were incubated with 100 pM TGF-β1 for 72 hours, kinase inhibitors were added, and incubation was continued for an additional 24 hours. F-actin was visualized by staining with Texas Red-phalloidin. Cells were viewed with an oil-objective lens at a 630× magnification. mTEC-KO cells were (A) untreated or treated with (B) 100 pM TGF-β1 for 72 hours followed by (C-E) single kinase inhibitor or (F-G) SB431542 plus a second kinase inhibitor. Single kinase inhibitors and concentrations were as follows: (C) 10 μM SB431542, (D) 10 μM SB203580, and (E) 10 μM Y27632. Combinations of kinase inhibitors were 10 μM SB431542 with (F) 10 μM SB203580 and (G) 10 μM Y27632. White arrows point to stress fibers. [file 1471-2121-10-94-S1.PDF]

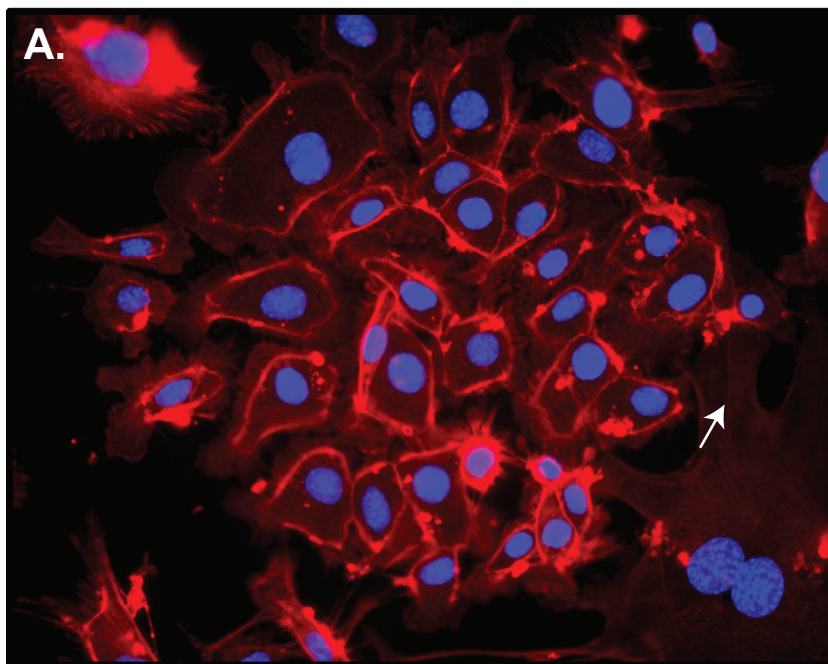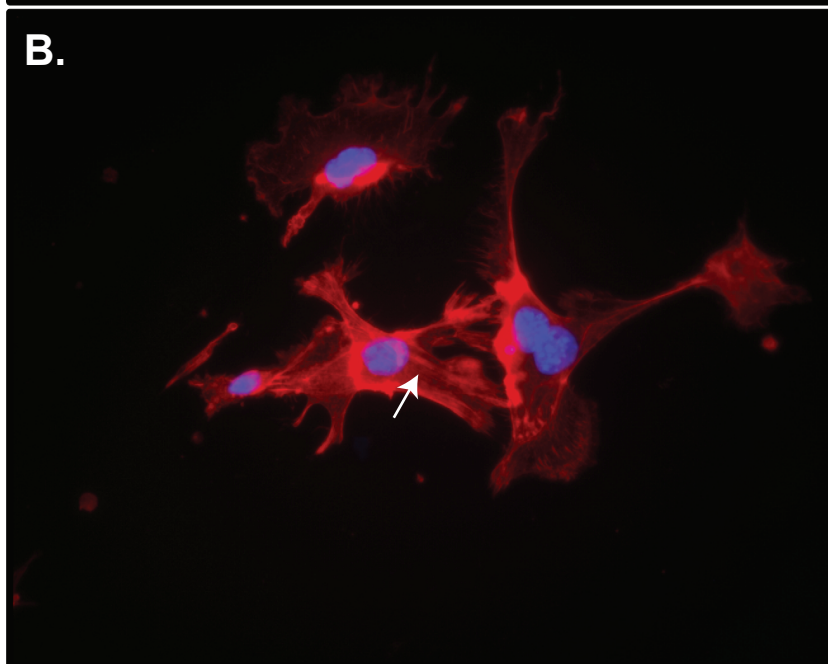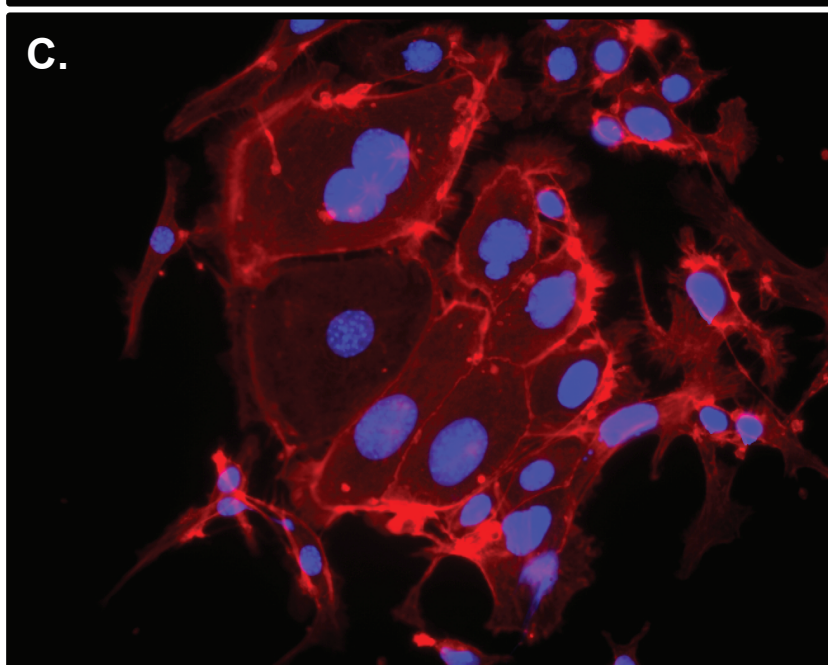

Supplement: Additional File 2 — A combination of TβRI inhibitor and a ROCK inhibitor is required to reverse EMT in mTEC-WT cells. mTEC-WT cells were incubated with 100 pM TGF-β1 for 72 hours, kinase inhibitors were added, and incubation was continued for an additional 24 hours. F-actin was visualized by staining with Texas Red-phalloidin. mTEC-WT cells were (A) untreated or treated with (B) 100 pM TGF-β1 followed by (C) 10 μM SB431542 plus 10 μM Y27632. White arrows point to stress fibers. [file 1471-2121-10-94-S2.PDF]

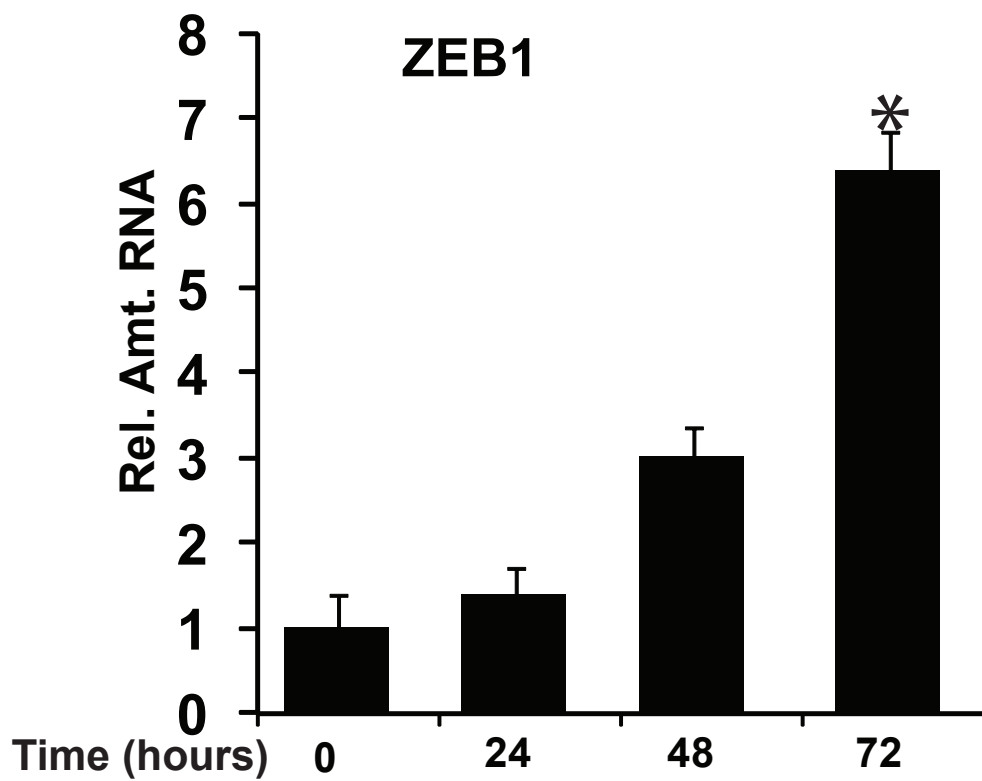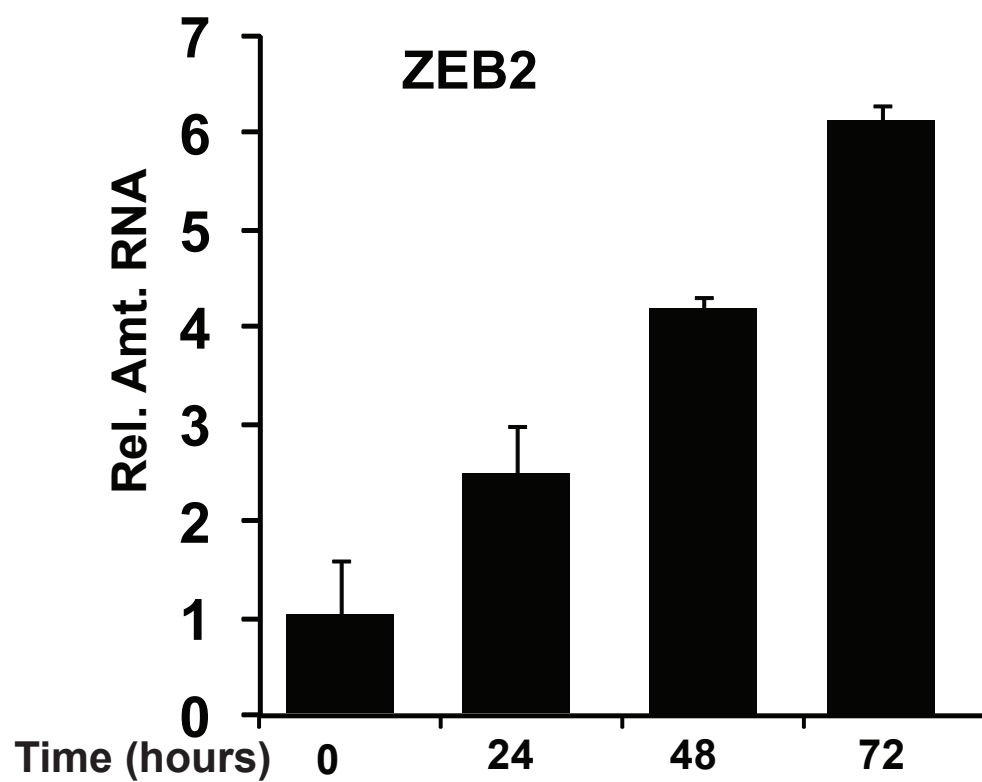

Supplement: Additional File 3 — TGF-β1 induces ZEB1 and ZEB2 RNA accumulation in mTEC-KO cells. mTEC-KO cells were incubated for the times indicated with 100 pM TGF-β1. Cells were harvested and assayed by quantitative RT-PCR for ZEB1 and ZEB2 RNA. Data shown are means + S.E.M.s of two experiments performed in triplicate. Asterisk (*) indicates significant difference (P < 0.05, n = 6). [file 1471-2121-10-94-S3.PDF]
